# Lamina Cribrosa Configurations in Highly Myopic and Non-Highly Myopic Eyes: The Beijing Eye Study

**DOI:** 10.1167/iovs.65.8.28

**Published:** 2024-07-18

**Authors:** Yingxiang Han, Xiaofei Wang, Can Can Xue, Jost B. Jonas, Ya Xing Wang

**Affiliations:** 1 Laboratory for Biomechanics and Mechanobiology of Ministry of Education, Beijing Advanced Innovation Center for Biomedical Engineering, School of Biological Science and Medical Engineering, Beihang University, Beijing, China; 2 Singapore Eye Research Institute, Singapore National Eye Centre, Singapore; 3 Rothschild Foundation Hospital, Institut Français de Myopie, Paris, France; 4 Beijing Institute of Ophthalmology, Beijing Tongren Hospital, Capital Medical University, Beijing Ophthalmology and Visual Sciences Key Laboratory, Beijing, China

**Keywords:** high myopia (HM), optic nerve head (ONH), lamina cribrosa depth (LCD), lamina cribrosa tilt (LCT), optical coherence tomography (OCT)

## Abstract

**Purpose:**

The purpose of this study was to examine characteristics of lamina cribrosa (LC) configuration in highly myopic (HM) eyes.

**Methods:**

Participants from the Beijing Eye Study 2011, free of optic nerve or retinal diseases, were randomly selected to examine LC depth (LCD) and LC tilt (LCT) using three-dimensional optical coherent tomography images of the optic nerve head (ONH). LCD and LCT were measured as the distance and angle between the LC plane with two reference planes, including the Bruch's membrane opening (BMO) plane and the peripapillary sclera (PPS) plane, respectively. Each parameter was measured in both horizontal and vertical B-scans.

**Results:**

The study included 685 individuals (685 eyes) aged 59.6 ± 7.7 years, including 72 HM eyes and 613 non-HM eyes. LCD measurements showed no significant differences between HM eyes and non-HM eyes in both horizontal (LCD-BMO = 421.83 ± 107.86 µm for HM eyes vs. 447.24 ± 104.94 µm for non-HM eyes, *P* = 0.18; and LCD-PPS = 406.39 ± 127.69 µm vs. 394.00 ± 101.64 µm, *P* = 1.00) and vertical directions (LCD-BMO = 435.78 ± 101.29 µm vs. 450.97 ± 106.54 µm, *P* = 0.70; and LCD-PPS = 401.62 ± 109.9 µm vs. 379.85 ± 110.35 µm, *P* = 0.35). However, the LCT was significantly more negative (tilted) in HM eyes than in non-HM eyes horizontally (LCT-BMO = −4.38 ± 5.94 degrees vs. −0.04 ± 5.86 degrees, *P* < 0.001; and LCT-PPS = −3.16 ± 5.23 degrees vs. −0.94 ± 4.71 degrees, *P* = 0.003), but not vertically (*P* = 1.00).

**Conclusions:**

Although LCD did not differ significantly between HM and non-HM eyes, LCT was more negative in HM eyes, suggesting that the temporal or inferior side of the LC was closer to the reference plane. These findings provide insights into morphological and structural changes in the LC and ONH between HM and non-HM eyes.

Glaucoma is one of the leading causes of irreversible blindness worldwide,^1^ and high myopia (HM) has been identified as a significant independent risk factor for glaucoma, exhibiting a dose-dependent relationship.^2^^,^^3^ The likelihood of developing open-angle glaucoma (OAG) increased from 1.50 to 4.14 as myopia severity shifted from low myopia to high myopia, with a sharper rise in glaucoma risk at a higher degree of myopia.^4^ However, the specific mechanisms underlying this association have remained unclear yet. In HM, the optic nerve head (ONH) undergoes major stretching and deformations, including but not restricted to the enlargement of Bruch’s membrane opening (BMO), elongation of the peripapillary scleral flange, and changes in the laminar cribrosa (LC), such as thinning and elongation.^5^^–^^7^ These marked morphological changes in the ONH lead to alterations in its biomechanical properties, which may partially explain the increased susceptibility to glaucomatous damage in HM.^4^

The LC is considered to be the site where glaucomatous damage to retinal ganglion cell axons occurs.^8^^–^^10^ Several studies have made efforts to address the issue by analyzing the morphological characteristics of the LC. Previous research has shown that the LC was histologically thinner in HM.^6^^,^^7^ It has been suggested that a thinner LC may be associated with increased susceptibility to glaucomatous damage due to the factors like intraocular pressure and intracranial pressure, because a thinner LC correlates with a more pronounced gradient of the trans-lamina cribrosa pressure difference.^6^^,^^11^^–^^13^ Yun et al. classified 210 healthy eyes into 3 groups based on axial length: long, middle, and short. They found that the lamina cribrosa depth (LCD) did not differ among different groups.^14^ The optic disc tilt is a signature in HM eyes and has been studied in several studies.^15^^–^^17^ Both the depth and the tilt of the LC, in conjunction with other myopia-related changes such as the development and enlargement of parapapillary gamma zone, may alter the biomechanical environment of the retinal ganglion cell axons when passing through the LC. This alteration may contribute to increased susceptibility of glaucoma. Although LC configurations have already been studied in several previous investigations, these studies predominantly utilized horizontal optical coherence tomography (OCT) B-scans, rather than capturing the overall configuration of the ONH. Due to the imaging challenges associated with the LC, most studies have focused on the general ONH structure rather than specifically on the LC.^18^^,^^19^ Furthermore, most prior research on the LC has involved hospital-based recruitment of participants, was restricted to relatively small cohorts, and relied on only a single OCT section, thus lacking on comprehensive data.

In view of the importance of the knowledge of the LC architecture for a better understanding of optic neuropathy, we therefore conducted this study, utilizing the three-dimensional OCT B-scans of the ONH from the participants of the population-based Beijing Eye Study, and examined the LC features, encompassing depth and tilt, in HM eyes and non-HM eyes.

## Methods

### Subject Recruitment

The Beijing Eye Study is a population-based study performed in the Greater Beijing region. The inclusion criteria were an age of 50 years or older in the year 2011, with 3468 individuals participating. The study design and study population have been described in detail previously.^20^^,^^21^ The study was approved by the Medical Ethics Committee of the Beijing Tongren Hospital and adhered to the tenets of the Declaration of Helsinki. Written informed consent was obtained from all participants.

Comprehensive ophthalmic examinations were conducted, including automatic refractometry, measurement of best corrected visual acuity (BCVA) and IOP, and digital photography of the cornea, lens, macula, optic disc, and macula (CR6-45MM; Conon Inc., Tokyo, Japan).^22^ The axial length was measured using optical low-coherence reflectometry (Lenstar 900; Optical Biometer, Haag-Streit, Koeniz, Switzerland). Spectral-domain OCT (Spectralis, Heidelberg Engineering, Heidelberg, Germany) with enhanced depth imaging modality was performed after pupillary dilation. The complete ONH imaging protocol consisted of 6 radial B-scans, each separated by 30 degrees, with each B scan comprising 512 A-scans, and an average of 100 repetitions.

For the present study, we randomly selected a subgroup of non-HM participants. The inclusion criterion was a BCVA of 20/25 or better for eyes with a refractive error (spherical equivalent) ranging between +1.00 and −4.00 diopters, and a BCVA of 20/33 or better for eyes with a refractive error of less than −4.00 diopters. All eligible HM participants were selected, with definition of a spherical equivalent of less than −6.00 diopters or an axial length greater than 26.5 mm. Only the right eyes were selected for the present study. We excluded all eyes with any types of retinal disease or optic neuropathy, including any type of glaucoma, diabetic retinopathy, status after ocular trauma or retinal detachment, retinal vein occlusions, age-related macular degeneration, or any other maculopathy.

### OCT Images Processing and Analysis

Raw OCT volumes were further enhanced using adaptive compensation to increase the visibility of the anterior surfaces of the peripapillary sclera and the LC.^23^^,^^24^ The horizontal and vertical B-scans running through the center of the optic disc were analyzed with custom-written MATLAB (MathWorks, Inc., Natick, MA, USA) algorithms, which has been used in our previous studies.^25^^–^^27^

**Figure 1.**
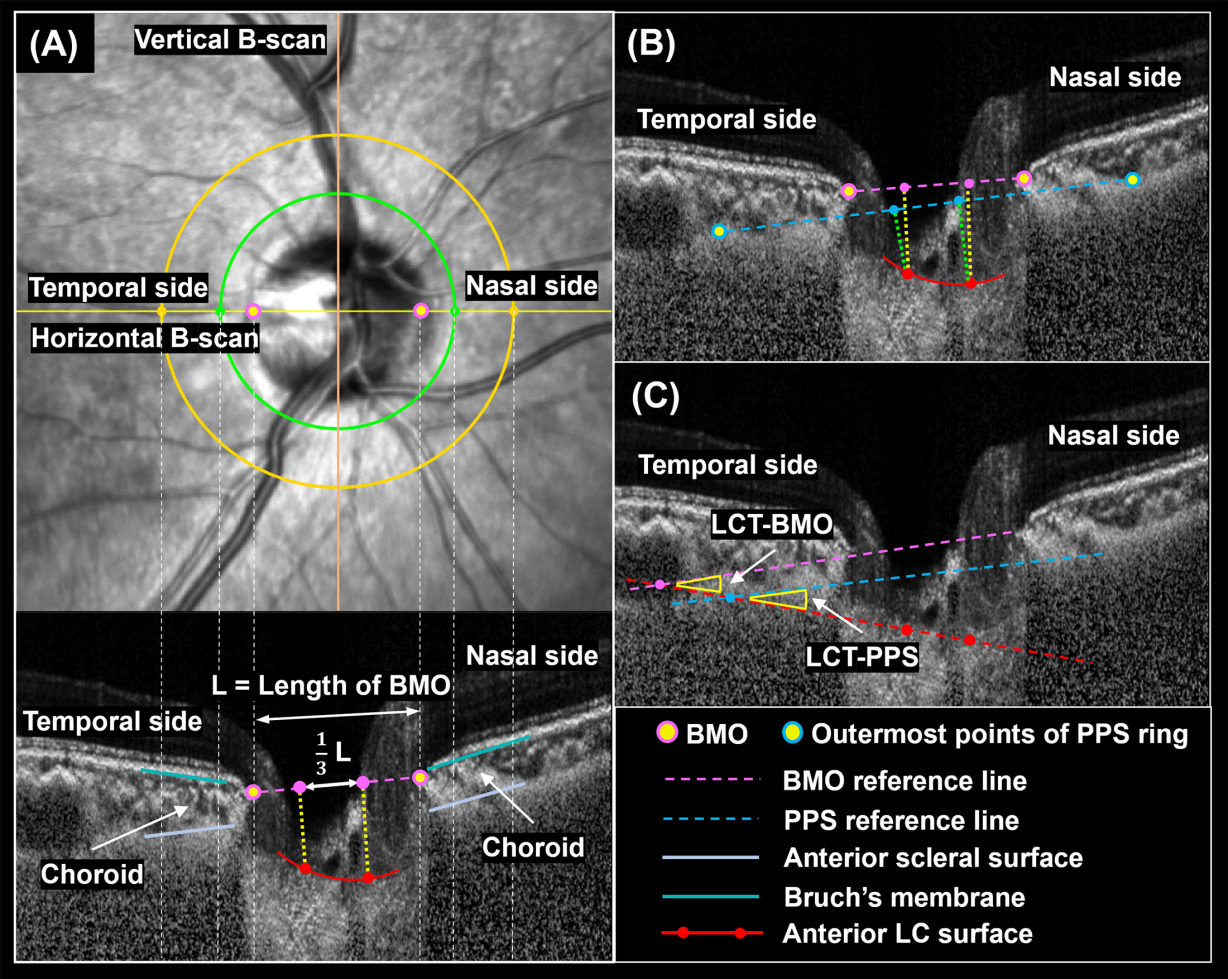
(**A**) Manually marked Bruch's membrane and Bruch's membrane opening (BMO), peripapillary sclera (PPS) and lamina cribrosa (LC). The peripapillary choroidal thickness was measured as the average thickness within the 1200 to 1800 µm ring region. (**B**) BMO and PPS reference planes, LCD-BMO and LCD-PPS. (**C**) LCT-BMO and LCT-PPS.

Identification of key structures of the ONH has been described in detail elsewhere.^26^ Briefly, the anterior surfaces of the sclera and LC were defined by a sharp increase in axial signal intensity. The BMO was defined as the endpoint of BM or of the complex of BM and the retinal pigment epithelium layer. The BMO was first manually marked and a peripapillary ring was defined from the center of the BMO with an inner and outer radius of 1200 µm and 1800 µm, respectively (Fig. 1). BM and the anterior surfaces of the peripapillary sclera (PPS) within the ring were then manually delineated. The anterior surface of the LC was also manually delineated.

The BMO reference plane was defined as the line connecting the two BMO points on either side of the ONH (see Fig. 1). The PPS reference plane was defined as the line connecting the outermost points of the PPS ring. BMO provides a relatively fixed internal boundary reference, whereas PPS provides a more external boundary reference. Using two reference planes enhances the accuracy and reliability of the measurements. Additionally, from a morphological perspective, the choroidal layer lies between the BMO reference plane and the anterior surface of the LC. Therefore, its thickness may influence the LC depth or tilt measurements. Using these delineations, the MATLAB algorithm calculated the following parameters automatically.

#### Lamina Cribrosa Depth

The perpendicular distance of the central anterior LC surface to the reference plane (BMO or PPS reference plane) was defined as the LCD. Central LC was determined as the portion within the central one-third of the diameter of the BMO. The average LCD of the central LC was noted as the mean LCD from each reference plane (see Fig. 1).

#### Lamina Cribrosa Tilt

The line connecting the two endpoints of the central LC was defined as the LC plane (see Fig. 1). The angle between the LC plane and each reference plane was defined as the LCT. In the horizontal B-scan, a negative LCT value indicated that the temporal side of the LC was higher than the nasal side with respect to the BMO or PPS reference plane. This meant that the temporal LC was closer to the reference plane (BMO or PPS) than the nasal LC. Similarly, in the vertical B-scan, a negative LCT indicated that the inferior side of the LC was higher than the superior side with respect to the reference planes. This meant that the inferior LC was closer to the reference plane than the superior LC. With a greater negative LCT value, the BMO and PPS on the temporal or inferior side showed a more pronounced backward tilt. To further analyze the LCT, we also computed the distance between the endpoints of the central LC (one third of the whole LC) and the reference planes. This allowed us to more precisely assess the spatial relationship between the LC and the reference planes.

#### Choroidal Thickness

Choroid thickness (ChT) was defined as the average distance between the BM and the anterior surface of the PPS within the PPS ring on both sides (see Fig. 1).

#### Disc-Fovea Distance

The disc-fovea distance was the distance from the fovea to the center of the optic disc on the fundus photograph.^28^

### InterObserver Repeatability of Measurements

We further assessed the repeatability of all measurements using a subset of OCT images from 20 randomly selected individuals, independently evaluated by 2 graders. The interclass correlation coefficients (ICCs) for these measurements were calculated.

### Statistical Analysis

We used the mean ± standard deviations to describe the demographic and ocular characteristics in each group. Student’s *t*-tests with Bonferroni correction were used to determine the statistical significance of differences between both groups. Correlations of measured parameters (LCD and LCT) with demographic parameters and ocular parameters were assessed by univariate and multivariable linear regression analysis. Variables with a *P* value less than 0.2 in the univariate analysis were included in the multivariate regression analysis. A *P* value of less than 0.05 was considered statistically significant. Statistics were performed in Python version 3.8 with packages of Pingouin 0.5, Pandas 1.3, and NumPy 1.2.0.

## Results

### Subjects and Ocular Parameters

A total of 685 eyes from 685 individuals (404 women, 59.0%) were included in the study with a mean age of 59.6 ± 7.7 (range = 50–90 years) years and a mean axial length of 23.6 ± 1.3 mm (range = 20.9–29.2 mm). Among them, 72 eyes were HM whereas 613 were non-HM (Table 1).

**Table 1.** Demographic Characteristics and Eye Parameters of the Subjects, and Comparisons Between Highly Myopic Eyes and Non-Highly Myopic Eyes

Variables	All Subjects, *n* = 685	Non-Highly Myopic Eyes, *n* = 613	Highly Myopic Eyes, *n* = 72	*P* Value
	Mean ± SD	Mean ± SD	Mean ± SD	
Age, y	59.6 ± 7.6	59.0 ± 7.3	64.3 ± 9.0	**<** **0.001**
Gender (female, count)	404 (0.659)	366 (0.597)	38 (0.528)	0.811
VA, unit	0.7 ± 0.3	0.7 ± 0.3	0.2 ± 0.1	**<** **0.001**
IOP, mm Hg	14.6 ± 2.6	14.6 ± 2.5	14.9 ± 2.9	1.00
CCT, µm	532.1 ± 32.6	531.0 ± 31.7	541.0 ± 38.7	0.137
ACD, mm	2.6 ± 0.4	2.5 ± 0.4	2.9 ± 0.5	**<** **0.001**
BMI, kg/m^2^	25.5 ± 3.7	25.6 ± 3.7	24.9 ± 3.4	0.349
ChT, µm	140.4 ± 51.9	144.0 ± 51.7	107.7 ± 41.4	**<** **0.001**
Axial length, mm	23.6 ± 1.3	23.3 ± 0.9	26.3 ± 1.4	**<** **0.001**
Spherical equivalent, diopters	−1.1 ± 2.5	−0.5 ± 1.5	−6.3 ± 3.1	**<** **0.001**
Disc-fovea distance, mm	4.9 ± 0.3	4.9 ± 0.3	4.9 ± 0.5	1.00
LCD in horizontal direction				
LCD-BMO, µm	444.6 ± 105.5	447.2 ± 104.9	421.8 ± 107.9	0.184
LCD-PPS, µm	395.3 ± 104.6	394.0 ± 101.6	406.4 ± 127.7	1.00
LCD in vertical direction				
LCD-BMO, µm	449.4 ± 106.0	451.0 ± 106.5	435.8 ± 101.3	0.703
LCD-PPS, µm	382.1 ± 110.4	379.8 ± 110.4	401.6 ± 109.9	0.346
LCT in horizontal direction				
LCT-BMO, µm	−0.5 ± 6.0	−0.0 ± 5.9	−4.4 ± 5.9	**<** **0.001**
LCT-PPS, µm	−1.2 ± 4.8	−0.9 ± 4.7	−3.2 ± 5.2	**0.003**
LCT in vertical direction				
LCT-BMO, µm	−1.4 ± 5.4	−1.1 ± 5.4	−4.1 ± 4.1	**<** **0.001**
LCT-PPS, µm	−3.2 ± 3.8	−3.2 ± 3.8	−3.2 ± 3.6	1.00

ACD, anterior chamber depth; CCT, central corneal thickness; ChT, choroidal thickness; SD, standard deviation; VA, visual acuity; BMI, body mass index.

As compared with the non-HM group, the HM group had an older age (59.0 ± 7.3 years vs. 64.3 ± 9.0 years, *P* < 0.001) and longer axial length (23.3 ± 0.9 mm vs. 26.3 ± 1.4 mm, *P* < 0.001), however, both groups did not differ significantly in sex (*P* = 0.81) and IOP (*P* = 1.00; see Table 1).

The mean LCD-BMO and LCD-PPS was 445 ± 106 µm and 395 ± 105 µm in the horizontal direction, and 449 ± 106 µm and 382 ± 110 µm in the vertical direction, respectively. When comparing the LCD between HM and non-HM, there was no significant difference either for the LCD-BMO (horizontal: *P* = 0.18; and vertical: *P* = 0.70) or the LCD-PPS (horizontal: *P* = 1.00; and vertical: *P* = 0.35; see Table 1). The association between LCD and demographic or ocular parameters are presented in Table 2 (LCD-BMO) and Supplementary Table S1 (LCD-PPS). After multivariable analysis, LCD was not significantly related with age and axial length. It was associated with a higher IOP, a thicker choroid and a higher body mass index as measured in both directions (*P* < 0.05). Additionally, men had a larger LCD than women in the vertical direction, for both the LCD-BMO (*P* = 0.010) and LCD-PPS (*P* = 0.003).

**Table 2.** Univariate and Multivariate Analysis of Demographic and Ocular Parameters in Relation to LCD-BMO Using Linear Regression Model

	Horizontal Direction						Vertical Direction					
	Univariable			Multivariable			Univariable			Multivariable		
Variables	β Coefficient	95% CI	*P* Value	β Coefficient	95% CI	*P* Value	β Coefficient	95% CI	*P* Value	β Coefficient	95% CI	*P* Value
**Age, y**	−1.886	(−3.03 to −0.74)	**0.001**	0.563	(−0.56 to 1.68)	0.324	−1.704	(−2.86 to −0.55)	**0.004**	0.655	(−0.45 to 1.76)	0.246
**Gender (ref: female)**	10.459	(−6.77 to 27.69)	0.234				14.778	(−2.49 to 32.05)	0.093	20.165	(4.78 to 35.55)	**0.010**
**IOP, mm Hg**	3.964	(0.68 to 7.25)	**0.018**	3.599	(0.63 to 6.57)	**0.018**	3.793	(0.49 to 7.09)	**0.024**	3.252	(0.32 to 6.18)	**0.030**
**CCT, µm**	−0.062	(−0.32 to 0.2)	0.638				−0.031	(−0.29 to 0.23)	0.815			
**BMI, kg/m^2^**	3.503	(1.23 to 5.77)	**0.003**	2.924	(0.86 to 4.99)	**0.006**	3.00	(0.72 to 5.28)	**0.01**	2.751	(0.72 to 4.79)	**0.008**
**ChT, µm**	0.892	(0.75 to 1.04)	**<0.001**	0.891	(0.73 to 1.05)	**<0.001**	0.931	(0.79 to 1.08)	**<0.001**	0.948	(0.79 to 1.1)	**<0.001**
**Disc-fovea distance, mm**	−38.390	(−68.66 to −8.12)	**0.013**	−13.602	(−41.09 to 13.89)	0.332	−41.202	(−71.55 to −10.85)	**0.008**	−15.039	(−42.17 to 12.09)	0.277
**Axial length, mm**	−9.213	(−16.22 to −2.21)	**0.01**	−0.869	(−7.42 to 5.68)	0.794	−4.579	(−11.64 to 2.48)	0.203			

BMI, body mass index; CCT, central corneal thickness; 95% CI, 95% confidence interval.

The *P* values in bold represent statistical significance.

The mean LCT-BMO and LCT-PPS was −0.5 ± 6.0 degrees and −1.2 ± 4.8 degrees in the horizontal direction, and −1.4 ± 5.4 degrees and −3.2 ± 3.8 degrees in the vertical direction, respectively. The association between LCT and demographic or ocular parameters are presented in Table 3 (LCT-BMO) and Supplementary Table S2 (LCT-PPS). In multivariable analysis of the associations of the LCT-BMO, a more horizontally tilted lamina (the temporal BMO with a more pronounced backward displacement) was associated with a longer axial length (*P* < 0.001) and a longer disc-fovea distance (*P* < 0.001), whereas it was not significantly associated with age, sex or IOP (*P* > 0.05). For a more vertically tilted lamina (the inferior BMO with a more pronounced backward displacement), the LCT-BMO was related only with a longer axial length (*P* = 0.002). In addition, the influencing factors and trends of LCD-PPS were the same as those of LCD-BMO measured in the horizontal direction. In the vertical direction, no factors were significantly associated with the LCT-PPS (see Supplementary Table S2).

**Table 3.** Univariate and Multivariate Analysis of Demographic and Ocular Parameters in Relation to LCT-BMO Using Linear Regression Model

	Horizontal Direction						Vertical Direction					
	Univariable			Multivariable			Univariable			Multivariable		
Variables	β Coefficient	95% CI	*P* Value	β Coefficient	95% CI	*P* Value	β Coefficient	95% CI	*P* Value	β Coefficient	95% CI	*P* Value
**Age (year)**	−0.063	(−0.13 to 0.0)	0.06	−0.026	(−0.09 to 0.04)	0.423	−0.007	(−0.07 to 0.05)	0.824			
**Gender (ref: female)**	−1.765	(−2.73 to −0.8)	**<0.001**	−0.680	(−1.65 to 0.29)	0.17	−1.177	(−2.07 to −0.28)	**0.01**	−0.717	(−1.65 to 0.21)	0.131
**IOP (mmHg)**	−0.140	(−0.33 to 0.05)	0.143	−0.139	(−0.34 to 0.06)	0.179	−0.032	(−0.2 to 0.14)	0.712			
**CCT (µm)**	−0.011	(−0.03 to 0.0)	0.15	0.004	(−0.01 to 0.02)	0.616	−0.005	(−0.02 to 0.01)	0.487			
**BMI (kg/m^2^)**	0.084	(−0.05 to 0.21)	0.205				0.024	(−0.1 to 0.14)	0.693			
**ChT (µm)**	0.004	(−0.0 to 0.01)	0.356				−0.003	(−0.01 to 0.01)	0.448			
**Disc-Fovea Distance (mm)**	−3.614	(−5.32 to −1.91)	**<0.001**	−3.946	(−5.58 to −2.32)	**<0.001**	−0.760	(−2.34 to 0.82)	0.346			
**Axial Length (mm)**	−1.443	(−1.83 to −1.06)	**<0.001**	−1.367	(−1.77 to −0.96)	**<0.001**	−0.702	(−1.06 to −0.34)	**<0.001**	−0.612	(−0.99 to −0.23)	**0.002**

Significant differences in LCTs were observed between HM eyes and non-HM eyes. In HM eyes, the LCT-BMO was markedly lower than in non-HM eyes, both horizontally (−4.4 ± 5.9 degrees vs. −0.0 ± 5.9 degrees, *P* < 0.001) and vertically (−4.1 ± 4.1 degrees vs. −1.1 ± 5.4 degrees, *P* < 0.001; see Table 1). Conversely, the difference in LCT-PPS between HM and non-HM eyes was significant only in the horizontal direction (−3.2 ± 5.2 degrees vs. 0.9 ± 4.7 degrees, *P* = 0.003), but not vertically (−3.2 ± 3.6 degrees vs. −3.2 ± 3.8 degrees, *P* = 1.00).

**Figure 2.**
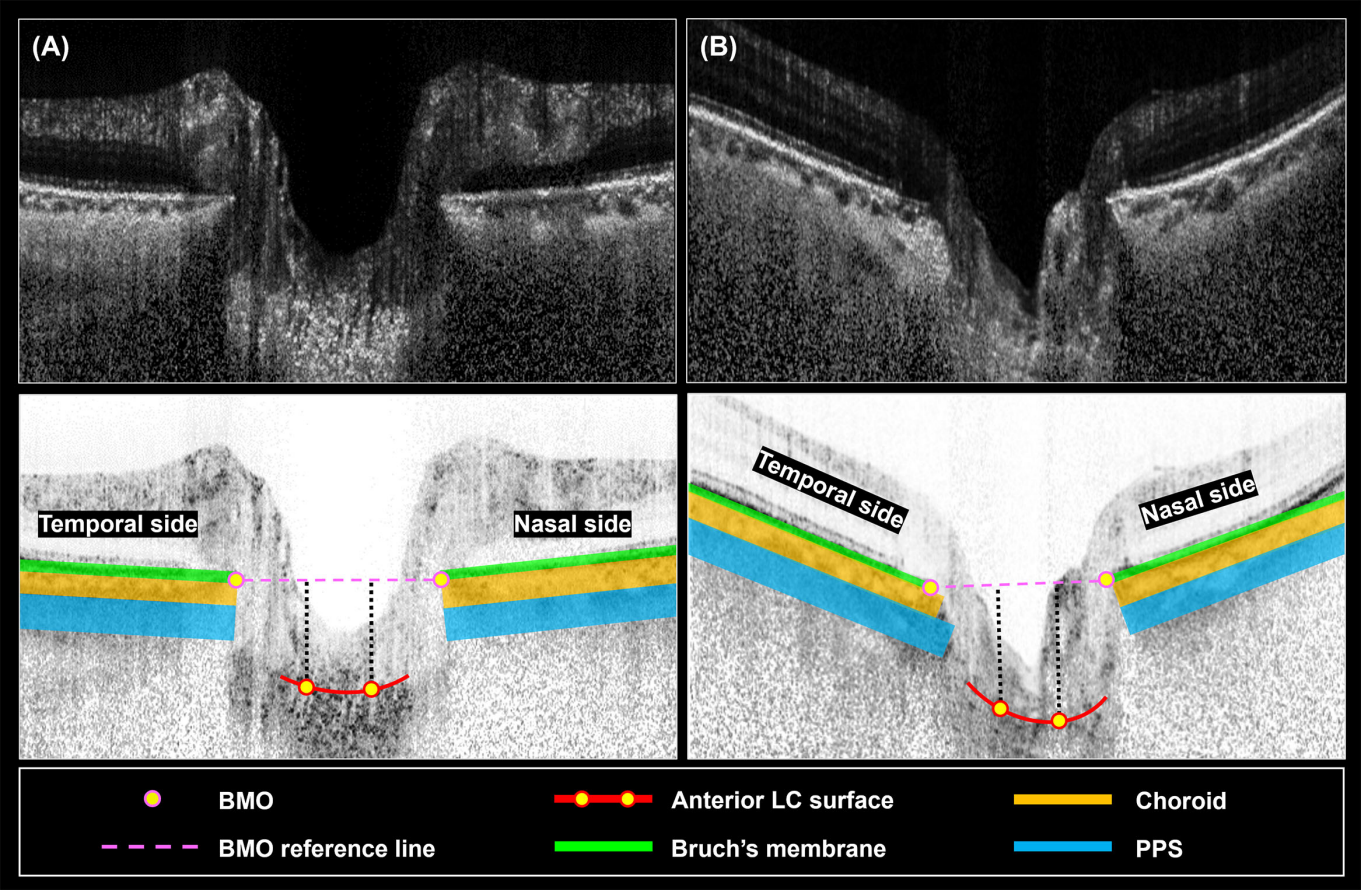
Schematic illustration showing the LCT-BMO in the horizontal direction with different spherical equivalent (right eyes). (**A**) Non-HM subject (OD), aged 61 years, spherical equivalent of 0.25 diopters, LCT of −0.5 degrees. (**B**) HM subject (OD), aged 61 years, spherical equivalent of −6.88 diopters, LCT of −5.8 degrees.

Although the central LCDs did not exhibit a substantial disparity between HM and non-HM, there was a notable difference in the configuration of the anterior surface of the LC. Specifically, the lamina tilted further away from the BMO/PPS plane on the nasal side, whereas conversely, the lamina displayed a tendency approaching these planes on the temporal side. Likewise, in the vertical scan, the LC tended to expand further away from the BMO plane in the superior region, while drawing closer to the plane inferiorly (*P* = 0.003; Fig. 2).

### InterObserver Repeatability of Image Segmentation

The ICCs for LCD-BMO, LCD-PPS, LCT-BMO, and LCT-PPS were 0.942, 0.968, 0.925, and 0.896, respectively, showing excellent consistency between 2 graders. Any discrepancies between the two observations were adjudicated by two senior ophthalmologists (authors X.W. and Y.X.W.) to ensure accuracy and consistency.

## Discussion

In this study, we conducted a comparative analysis of the depth and tilt of the anterior LC surface between healthy eyes and HM eyes in a population-based recruited cohort of Chinese people. Our finding revealed no significant differences between the non-HM group and the HM group with respect to the LCDs (either LCD-BMO or LCD-PPS), determined in the horizontal and vertical directions, as well as LCT-PPS measured in the vertical direction. The LCTs (either LCT-BMO or LCT-PPS) in the horizontal plane of HM eyes differed significantly from the LCTs in non-HM eyes. The LCTs determined in the horizontal direction of HM eyes were significantly smaller (a negative value) than those of non-HM eyes, indicating a closer proximity of the temporal LC to the BMO and the PPS reference planes (a more tilted shape).

The LCDs of non-HM eyes in the horizontal direction (LCD-BMO: 447.2 ± 104.9 µm; LCD-PPS: 394.0 ± 101.6 µm; see Table 1) were compared with those obtained from previous studies. Tun et al.^25^ measured the LCD-BMO and LCD-PPS of 628 Chinese subjects in Singapore and found that the median values for LCD-BMO and LCD-PPS were 426 µm and 366 µm, respectively. Luo et al.^29^ reported LCD-BMO values of 402 ± 91 µm and LCD-PPS values of 332 ± 96 µm in a multi-ethnic cohort. Another study of 150 healthy Korean individuals found an LCD-BMO of 402 ± 102 µm.^30^ The LCDs reported in this study were, on average, higher than those assessed in other studies, which might be attributed to differences in the ethnic background, demographic parameters, and measurement methods.

Previous studies have identified that glaucomatous eyes exhibited a significantly deeper optic cup with a larger LCD compared with non-glaucomatous eyes.^14^^,^^31^^,^^32^ It has been interpreted as a typical feature of glaucoma. However, no LCD deepening was found in HM eyes in the current study, with HM eyes and non-HM eyes not differing significantly in LCDs. In addition, axial length was not significantly associated with the LCD-BMO and LCD-PPS in both horizontal direction and vertical direction. Some previous studies suggested that during the process of axial elongation and myopia, LC gradually moved to the nasal side, leading to a change in the peripapillary structures.^33^^–^^38^ This might indicate that during axial elongation, the deep tissues of the ONH and LC undergo a movement in the horizontal rather than in the vertical or sagittal direction. It might suggest that the LCD deepening was not related to axial length and HM.

In this study, multivariate regression analysis showed that deeper LCDs were associated with higher IOP. This finding agrees with observations made in previous studies which also found a positive correlation between IOP and LCDs in the context of glaucoma.^31^^,^^39^^,^^40^ Earlier studies noted that higher IOP led to a backward bending and deformation of the LC.^40^ Furthermore, Sigal et al.^41^^,^^42^ reported that the impact of IOP on the LCD was determined by the balance between the direct effect of the IOP pushing the LC posteriorly and the indirect effect of the IOP deforming and expanding the sclera, resulting in a lateral pulling of the LC and reduction of the LCD. Therefore, LCD changes with IOP were affected by the morphological and biomechanical characteristics of the ONH. For instance, a stiff sclera deformed little under elevated IOP, which limited the scleral expansion, allowing the LC to be displaced posteriorly by an IOP elevation.^41^^,^^43^

Although previous studies have shown that ChT was associated with the measurements of LCD-BMO, whereas readings of the LCD-PPS were not or less affected, we found that ChT was one of the main factors associated with both LCD-PPS and LCD-BMO.^44^ In a previous study based on a large number of healthy individuals, ChT was positively correlated with LCD-PPS and LCD-BMO, which is consistent with our results.^25^ In addition, our results showed that the LCD-PPS and LCD-BMO were deeper in men in the vertical direction, and this finding is similar to previous studies, which also reported a similar finding in the horizontal direction.^25^^,^^29^ In fact, sex-related differences, such as parapapillary retinal nerve fiber layer thickness distribution^45^ and visibility score,^46^ have been reported in many other ONH morphologic studies.

This study introduced LCT as a new parameter to quantify the local LC tilt in the scleral canal with reference to other ONH tissues. This approach differs from the use of parameters in previous studies that quantify the ONH tilt or torsion in terms of the global tilt of the entire ONH.^21^^,^^47^ Our findings indicated that LC deepening is not a specific feature of HM, whereas a more tilted LC (the temporal or inferior reference plane with a more pronounced backward displacement) was observed more marked in HM eyes than in non-HM eyes. Furthermore, in the multivariable regression analysis, axial length was not the main influencing factor for LCD but it was for LCT. In fact, LC thickness and elongation and parapapillary scleral morphology, but not LCD, are significantly different in HM and non-HM.^5^^,^^6^^,^^14^ Therefore, when investigating the influences of morphologic changes of the LC on the incidence and development of glaucoma in HM, the thinning, elongation, and tilt of the LC should be focused on rather than the depth of the anterior surface of the LC. The misalignment between the tilted LC caused by excessive axial elongation and the BMO/PPS could alter the trajectory of retinal ganglion cell axons passing through the LC pores and influence the biomechanical environment to which the retinal ganglion cell axons are exposed. Further studies are needed to investigate the influence of LC tilt on the mechanical response of LC and on the retinal ganglion cell axons.

In our study, both LCD-BMO and LCD-PPS, in either HM or non-HM eyes, were influenced by ChT, demonstrating the effectiveness of including both of these reference planes for measuring LCD. Although the depth of LC was found to be similar in the two groups, the LC in HM eyes was observed to be more tilted than in non-HM eyes with respect to the BMO reference plane, both horizontally and vertically. In contrast, the LCT-PPS differed only in the horizontal direction. Therefore, utilizing the BMO reference plane may better delineate the discrepancies in LCT between HM eyes and non-HM eyes. Furthermore, the closer distances of the LC to the BMO reference plane on the temporal side (horizontal direction) or inferior side (vertical direction) in HM eyes might indicate that the temporal or inferior BMO regions exhibited a more pronounced posterior displacement (see Fig. 2, Supplementary Table S3). The different distances could be caused by altered peripapillary structures with the process of axial length elongation and myopia,^37^^,^^38^ confirming the differences in LCT between HM and non-HM. Although this posterior displacement did not promote LCD deepening, it could alter the LC and its surrounding mechanical environment.

The study was subject to several limitations. First, the analysis was confined to the central horizontal and vertical B-scans to quantify LCD and LCT, which may have overlooked morphological nuances in other planes. Second, the inclusion of only non-glaucomatous limited our scope to HM and non-HM eyes, preventing insights from glaucoma-specific configurations in HM eyes. Further work is warranted to explore these configurations. Third, we did not measure the LC thickness in the current study due to the limited penetration depth of OCT, which compromised the reliability of such measurements.

Despite these, our study stands out as the first to use three-dimensional ONH segmentation in a population-based study, providing a detailed evaluation of the LC characteristics. This approach enabled a comprehensive understanding of specific LC configurations in HM eyes compared to non-HM eyes, highlighting position-specific changes in the LC configuration. By utilizing this method, we provided a semi three-dimensional perspective on the architecture of the LC, specifically enriching our understanding of the complexities of the LC morphology in varying ocular conditions. This enhanced visualization and analytical capacity enable the development of more precise and effective diagnostic protocols, facilitating earlier and more accurate differentiation between normal anatomic variations and pathological changes that may indicate an increased risk of glaucoma.

In summary, our population-based study utilized a three-dimensional analysis of the ONH to quantify the features of the LC, including LCD and LCT. The LCD showed no significant difference between HM eyes and non-HM eyes. The LCT was reduced (indicated by a negative value) in HM eyes when compared with non-HM eyes, with the temporal or inferior side of the LC positioned closer to the reference plane. These findings indicate that HM eyes exhibit a more pronounced LC tilt rather a deeper LC anterior surface, which could be associated with the structural and biomechanical changes in the ONH due to myopia. These observations may be helpful for assessing the morphological and structural alterations in the LC and other parts of the ONH and improve clinical assessments and interventions.

## Supplementary Material

Supplement 1

## References

[1] Quigley HA, Broman AT. The number of people with glaucoma worldwide in 2010 and 2020. *Br J Ophthalmol*. 2006; 90: 262–267. 16488940 10.1136/bjo.2005.081224 PMC1856963

[2] Xu L, Wang Y, Wang S, Wang Y, Jonas JB. High myopia and glaucoma susceptibility: the Beijing Eye Study. *Ophthalmology*. 2007; 114: 216–220. 17123613 10.1016/j.ophtha.2006.06.050

[3] Jonas JB, Wang YX, Dong L, Panda-Jonas S. High myopia and glaucoma-like optic neuropathy. *Asia Pac J Ophthalmol (Phila)*. 2020; 9: 234–238. 32459697 10.1097/APO.0000000000000288 PMC7299230

[4] Ha A, Kim CY, Shim SR, Chang IB, Kim YK. Degree of myopia and glaucoma risk: a dose-response meta-analysis. *Am J Ophthalmol*. 2022; 236: 107–119. 34648776 10.1016/j.ajo.2021.10.007

[5] Ren R, Wang N, Li B, et al. Lamina cribrosa and peripapillary sclera histomorphometry in normal and advanced glaucomatous chinese eyes with various axial length. *Invest Ophthalmol Vis Sci*. 2009; 50: 2175. 19387083 10.1167/iovs.07-1429

[6] Jonas JB, Berenshtein E, Holbach L. Lamina cribrosa thickness and spatial relationships between intraocular space and cerebrospinal fluid space in highly myopic eyes. *Invest Ophthalmol Vis Sci*. 2004; 45: 2660. 15277489 10.1167/iovs.03-1363

[7] Jonas JB, Wang YX, Dong L, Guo Y, Panda-Jonas S. Advances in myopia research anatomical findings in highly myopic eyes. *Eye Vis (Lond)*. 2020; 7: 45. 32905133 10.1186/s40662-020-00210-6 PMC7465809

[8] Quigley HA, Anderson DR. The dynamics and location of axonal transport blockade by acute intraocular pressure elevation in primate optic nerve. *Invest Ophthalmol*. 1976; 15: 606–616. 60300

[9] Quigley HA, Addicks EM, Green WR, Maumenee AE. Optic nerve damage in human glaucoma: II. The site of injury and susceptibility to damage. *Arch Ophthalmol*. 1981; 99: 635–649. 6164357 10.1001/archopht.1981.03930010635009

[10] Tan NY, Koh V, Girard MJ, Cheng CY. Imaging of the lamina cribrosa and its role in glaucoma: a review. *Clin Exp Ophthalmol*. 2018; 46: 177–188. 29214709 10.1111/ceo.13126

[11] Kim S, Sung KR, Lee JR, Lee KS. Evaluation of lamina cribrosa in pseudoexfoliation syndrome using spectral-domain optical coherence tomography enhanced depth imaging. *Ophthalmology*. 2013; 120: 1798–1803. 23622874 10.1016/j.ophtha.2013.02.015

[12] Park HYL, Jeon SH, Park CK. Enhanced depth imaging detects lamina cribrosa thickness differences in normal tension glaucoma and primary open-angle glaucoma. *Ophthalmology*. 2012; 119: 10–20. 22015382 10.1016/j.ophtha.2011.07.033

[13] Lee EJ, Kim TW, Kim M, Kim H. Influence of lamina cribrosa thickness and depth on the rate of progressive retinal nerve fiber layer thinning. *Ophthalmology*. 2015; 122: 721–729. 25433610 10.1016/j.ophtha.2014.10.007

[14] Yun SC, Hahn IK, Sung KR, Yoon JY, Jeong D, Chung HS. Lamina cribrosa depth according to the level of axial length in normal and glaucomatous eyes. *Graefes Arch Clin Exp Ophthalmol*. 2015; 253: 2247–2253. 26267752 10.1007/s00417-015-3131-y

[15] Tay E, Seah SK, Chan SP, et al. Optic disk ovality as an index of tilt and its relationship to myopia and perimetry. *Am J Ophthalmol*. 2005; 139: 247–252. 15733984 10.1016/j.ajo.2004.08.076

[16] Li Z, Guo X, Xiao O, et al. Optic disc features in highly myopic eyes: the ZOC-BHVI High Myopia Cohort Study. *Optom Vis Sci*. 2018; 95: 318–322. 29561500 10.1097/OPX.0000000000001200

[17] Zhang F, Liu X, Wang Y, et al. Characteristics of the optic disc in young people with high myopia. *BMC Ophthalmol*. 2022; 22: 477. 36482327 10.1186/s12886-022-02719-x PMC9730557

[18] Lee KM, Lee EJ, Kim TW. Lamina cribrosa configuration in tilted optic discs with different tilt axes: a new hypothesis regarding optic disc tilt and torsion. *Invest Ophthalmol Vis Sci*. 2015; 56: 2958. 25788647 10.1167/iovs.14-15953

[19] Hasegawa T, Akagi T, Hangai M, et al. Structural dissociation of optic disc margin components with optic disc tilting: a spectral domain optical coherence tomography study. *Graefes Arch Clin Exp Ophthalmol*. 2016; 254: 343–349. 26582160 10.1007/s00417-015-3210-0

[20] Xu J, Xu L, Du KF, et al. Subfoveal choroidal thickness in diabetes and diabetic retinopathy. *Ophthalmology*. 2013; 120: 2023–2028. 23697958 10.1016/j.ophtha.2013.03.009

[21] Zhang Q, Wang YX, Wei WB, Xu L, Jonas JB. Parapapillary beta zone and gamma zone in a healthy population: the Beijing Eye Study 2011. *Invest Ophthalmol Vis Sci*. 2018; 59: 3320–3329. 30025091 10.1167/iovs.18-24141

[22] Jiang R, Wang YX, Wei WB, Xu L, Jonas JB. Peripapillary choroidal thickness in adult Chinese: the Beijing Eye Study. *Invest Ophthalmol Vis Sci*. 2015; 56: 4045. 26114482 10.1167/iovs.15-16521

[23] Girard MJA, Strouthidis NG, Ethier CR, Mari JM. Shadow removal and contrast enhancement in optical coherence tomography images of the human optic nerve head. *Invest Ophthalmol Vis Sci*. 2011; 52: 7738–7748. 21551412 10.1167/iovs.10-6925

[24] Girard MJA, Tun TA, Husain R, et al. Lamina cribrosa visibility using optical coherence tomography: comparison of devices and effects of image enhancement techniques. *Invest Ophthalmol Vis Sci*. 2015; 56: 865–874. 25593025 10.1167/iovs.14-14903

[25] Tun TA, Wang X, Baskaran M, et al. Determinants of lamina cribrosa depth in healthy Asian eyes: the Singapore Epidemiology Eye Study. *Br J Ophthalmol*. 2021; 105: 367–373. 32434775 10.1136/bjophthalmol-2020-315840

[26] Tun TA, Wang X, Baskaran M, et al. Variation of peripapillary scleral shape with age. *Invest Ophthalmol Vis Sci*. 2019; 60: 3275–3282. 31369672 10.1167/iovs.19-26777 PMC6675518

[27] Xue CC, Wang X, Han YX, et al. Parapapillary gamma zone associated with increased peripapillary scleral bowing: the Beijing Eye Study 2011. *Br J Ophthalmol*. 2023; 107: 1665–1671. 36126108 10.1136/bjo-2022-321868 PMC10646846

[28] Jonas RA, Wang YX, Yang H, et al. Optic disc - fovea distance, axial length and parapapillary zones. The Beijing Eye Study 2011. Frishman L, ed. *PLoS One*. 2015; 10: e0138701. 26390438 10.1371/journal.pone.0138701 PMC4577126

[29] Luo H, Yang H, Gardiner SK, et al. Factors influencing central lamina cribrosa depth: a multicenter study. *Invest Ophthalmol Vis Sci*. 2018; 59: 2357. 29847642 10.1167/iovs.17-23456 PMC5939685

[30] Seo JH, Kim TW, Weinreb RN. Lamina cribrosa depth in healthy eyes. *Invest Ophthalmol Vis Sci*. 2014; 55: 1241–1251. 24474269 10.1167/iovs.13-12536

[31] Lee SH, Kim TW, Lee EJ, Girard MJA, Mari JM. Diagnostic power of lamina cribrosa depth and curvature in glaucoma. *Invest Ophthalmol Vis Sci*. 2017; 58: 755. 28146240 10.1167/iovs.16-20802

[32] Lee KM, Kim M, Oh S, Kim SH. Hemisphere opposite to vascular trunk deviation is earlier affected by glaucomatous damage in myopic high-tension glaucoma. Belghith A, ed. *PLoS One*. 2020; 15: e0233270. 32421695 10.1371/journal.pone.0233270 PMC7233594

[33] Kim YW, Choi JJ, Girard MJA, Mari JM, Choi DG, Park KH. Longitudinal observation of border tissue configuration during axial elongation in childhood. *Invest Ophthalmol Vis Sci*. 2021; 62: 10. 10.1167/iovs.62.4.10 PMC8039469 33825856

[34] Lee KM, Choung HK, Kim M, Oh S, Kim SH. Positional change of optic nerve head vasculature during axial elongation as evidence of lamina cribrosa shifting: Boramae Myopia Cohort Study Report 2. *Ophthalmology*. 2018; 125: 1224–1233. 29544962 10.1016/j.ophtha.2018.02.002

[35] Lee S, Heisler M, Ratra D, et al. Effects of myopia and glaucoma in the prelaminar neural canal and anterior lamina cribrosa using 1060-nm swept-source optical coherence tomography. *J Glaucoma*. 2023; 32: 48–56. 36584358 10.1097/IJG.0000000000002107 PMC10503542

[36] Lee KM, Ahn HJ, Kim M, Oh S, Kim SH. Offset of openings in optic nerve head canal at level of Bruch's membrane, anterior sclera, and lamina cribrosa. *Sci Rep*. 2021; 11: 22435. 34789748 10.1038/s41598-021-01184-8 PMC8599705

[37] Kim M, Kim SY, Lee KM, Oh S, Kim SH. Position of central vascular trunk and shape of optic nerve head in newborns. *Invest Ophthalmol Vis Sci*. 2019; 60: 3381. 31387114 10.1167/iovs.19-27363

[38] Kim M, Choung HK, Lee KM, Oh S, Kim SH. Longitudinal changes of optic nerve head and peripapillary structure during childhood myopia progression on OCT. *Ophthalmology*. 2018; 125: 1215–1223. 29550000 10.1016/j.ophtha.2018.01.026

[39] Lee EJ, Kim TW, Kim H, Lee SH, Girard MJA, Mari JM. Comparison between lamina cribrosa depth and curvature as a predictor of progressive retinal nerve fiber layer thinning in primary open-angle glaucoma. *Ophthalmol Glaucoma*. 2018; 1: 44–51. 32672632 10.1016/j.ogla.2018.05.007

[40] Wu J, Du Y, Li J, Fan X, Lin C, Wang N. The influence of different intraocular pressure on lamina cribrosa parameters in glaucoma and the relation clinical implication. *Sci Rep*. 2021; 11: 9755. 33963202 10.1038/s41598-021-87844-1 PMC8105377

[41] Sigal IA, Yang H, Roberts MD, et al. IOP-induced lamina cribrosa deformation and scleral canal expansion: independent or related? *Invest Ophthalmol Vis Sci*. 2011; 52: 9023. 21989723 10.1167/iovs.11-8183 PMC3231799

[42] Sigal IA, Ethier CR. Biomechanics of the optic nerve head. *Exp Eye Res*. 2009; 88: 799–807. 19217902 10.1016/j.exer.2009.02.003

[43] Grytz R, Meschke G, Jonas JB. The collagen fibril architecture in the lamina cribrosa and peripapillary sclera predicted by a computational remodeling approach. *Biomech Model Mechanobiol*. 2011; 10: 371–382. 20628781 10.1007/s10237-010-0240-8

[44] Vianna JR, Lanoe VR, Quach J, et al. Serial changes in lamina cribrosa depth and neuroretinal parameters in glaucoma: impact of choroidal thickness. *Ophthalmology*. 2017; 124: 1392–1402. 28461018 10.1016/j.ophtha.2017.03.048

[45] Jonas JB, Yan YN, Zhang Q, et al. Retinal nerve fibre layer thickness in association with gamma zone width and disc-fovea distance. *Acta Ophthalmol*. 2022; 100: 632–639. 35076179 10.1111/aos.15088

[46] Zhang Y, Xu L, Zhang L, Yang H, Wang YX, Jonas JB. Ophthalmoscopic assessment of the retinal nerve fiber layer. The Beijing Eye Study. Bui BV, ed. *PLoS One*. 2013; 8: e62022. 23637954 10.1371/journal.pone.0062022 PMC3639254

[47] Hosseini H, Nassiri N, Azarbod P, et al. Measurement of the optic disc vertical tilt angle with spectral-domain optical coherence tomography and influencing factors. *Am J Ophthalmol*. 2013; 156: 737–744.e1. 23891337 10.1016/j.ajo.2013.05.036

