# Supplementary material for: Lamina Cribrosa Configurations in Highly Myopic and Non-Highly Myopic Eyes: The Beijing Eye Study
Source: Invest Ophthalmol Vis Sci. 2024 Jul 18;65(8):28. doi: 10.1167/iovs.65.8.28 (PMC11262544; doi:10.1167/iovs.65.8.28)
Supplement: Supplement 1 [file iovs-65-8-28_s001.pdf]

Supplementary Materials

Table 1. Univariate and multivariate analysis of demographic and ocular parameters in relation to LCD-PPS using linear regression model.

Variables	Horizontal Direction						Vertical Direction					
	Univariable			Multivariable			Univariable			Multivariable		
	**β coefficient**	**95% CI**	**P value**	**β coefficient**	**95% CI**	**P Value**	**β coefficient**	**95% CI**	**P value**	**β coefficient**	**95% CI**	**P Value**
**Age (year)**	0.197	(-0.92 to 1.32)	0.73				-0.174	(-1.37 to 1.02)	0.774			
**Gender (ref: female)**	10.318	(-6.35 to 26.99)	0.225				21.684	(3.98 to 39.39)	**0.016**	25.918	(9.1 to 42.73)	**0.003**
**IOP (mmHg)**	3.759	(0.58 to 6.94)	**0.021**	3.418	(0.35 to 6.48)	**0.029**	4.134	(0.75 to 7.52)	**0.017**	3.501	(0.3 to 6.7)	**0.032**
**CCT (μm)**	-0.063	(-0.31 to 0.19)	0.619				0.002	(-0.26 to 0.27)	0.986			
**BMI (kg/㎡)**	2.126	(-0.08 to 4.33)	0.059	1.729	(-0.4 to 3.86)	0.111	1.886	(-0.47 to 4.24)	0.116	1.784	(-0.45 to 4.01)	0.117
**ChT (μm)**	0.523	(0.37 to 0.68)	**<0.001**	0.508	(0.35 to 0.66)	**<0.001**	0.667	(0.51 to 0.83)	**<0.001**	0.663	(0.5 to 0.82)	**<0.001**
**Disc-Fovea Distance (mm)**	-21.753	(-51.14 to 7.63)	0.147	-7.687	(-36.2 to 20.83)	0.597	-26.132	(-57.44 to 5.17)	0.102	-8.369	(-38.05 to 21.31)	0.58
**Axial Length (mm)**	-3.095	(-9.91 to 3.72)	0.373				2.008	(-5.26 to 9.27)	0.587			

CCT = Central corneal thickness;

ChT = Choroidal thickness.

Table 2. Univariate and multivariate analysis of demographic and ocular parameters in relation to LCD-PPS using linear regression model.

	Horizontal Direction						Vertical Direction					
Variables	Univariable			Multivariable			Univariable			Multivariable		
	**β coefficient**	**95% CI**	**P value**	**β coefficient**	**95% CI**	**P Value**	**β coefficient**	**95% CI**	**P value**	**β coefficient**	**95% CI**	**P Value**
**Age (year)**	-0.037	(-0.09 to 0.02)	0.173	-0.003	(-0.06 to 0.05)	0.926	0.032	(-0.01 to 0.07)	0.137	0.031	(-0.01 to 0.08)	0.181
**Gender (ref: female)**	-1.351	(-2.13 to -0.57)	**0.001**	-0.461	(-1.25 to 0.33)	0.252	-0.678	(-1.3 to -0.05)	**0.033**	-0.598	(-1.25 to 0.06)	0.073
**IOP (mmHg)**	-0.131	(-0.28 to 0.02)	0.088	-0.126	(-0.29 to 0.04)	0.134	-0.024	(-0.14 to 0.1)	0.696			
**CCT (μm)**	-0.009	(-0.02 to 0.0)	0.138	0.004	(-0.01 to 0.02)	0.588	-0.004	(-0.01 to 0.01)	0.396			
**BMI (kg/㎡)**	0.097	(-0.01 to 0.2)	0.067	0.035	(-0.07 to 0.14)	0.49	0.035	(-0.05 to 0.12)	0.407			
**ChT (μm)**	0.008	(0.0 to 0.02)	**0.027**	0.001	(-0.01 to 0.01)	0.843	-0.006	(-0.01 to 0.0)	0.06	-0.006	(-0.01 to 0.0)	0.082
**Disc-Fovea Distance (mm)**	-2.191	(-3.57 to -0.81)	**0.002**	-2.405	(-3.75 to -1.06)	**<0.001**	0.157	(-0.95 to 1.26)	0.78			
**Axial Length (mm)**	-1.183	(-1.49 to -0.88)	**<0.001**	-1.123	(-1.46 to -0.79)	**<0.001**	-0.207	(-0.46 to 0.05)	0.111	-0.221	(-0.5 to 0.05)	0.114

CCT = Central corneal thickness;

ChT = Choroidal thickness.

Table 3. Demographic characteristics and eye parameters of the subjects, and comparisons between highly myopic eyes and non-highly myopic eyes.

Variables	Non-Highly Myopic Eyes, n=613	Highly Myopic Eyes, n=72	P Value
	Mean±Standard deviations (SD)	Mean±SD	
**Horizontal Direction**			
LC-BMO-T (μm)	459.0±109.2	396.0±114.6	**<0.001**
LC-BMO-N (μm)	459.6±103.7	440.1±104.0	0.409
LC-PPS-T (μm)	380.1±102.8	384.2±134.2	>0.999
LC-PPS-N (μm)	388.9±101.7	417.0±124.9	0.21
**Vertical Direction**			
LC-BMO-I (μm)	474.5±117.6	413.5±100.6	**<0.001**
LC-BMO-S (μm)	485.7±109.4	456.9±105.3	0.094
LC-PPS-I (μm)	357.5±108.0	381.2±109.4	0.253
LC-PPS-S (μm)	389.7±113.6	414.9±116.9	0.258

LC-BMO / PPS-T = The distance between the temporal side of LC and the BMO / PPS reference plane;
LC-BMO/ PPS -N = The distance between the nasal side of LC and the BMO / PPS reference plane;
LC-BMO / PPS-I = The distance between the inferior side of LC and the BMO / PPS reference plane;
LC-BMO / PPS-S = The distance between the superior side of LC and the BMO / PPS reference plane.
